# Complex multimorbidity in hemodialysis patients: Study in a metropolitan region in Brazil

**DOI:** 10.1371/journal.pone.0303068

**Published:** 2024-05-16

**Authors:** Ana Cristina de Oliveira Soares, Glenda Blaser Petarli, Monica Cattafesta, Edson Theodoro dos Santos Neto, Luciane Bresciani Salaroli

**Affiliations:** Graduate Program in Collective Health, Federal University of Espírito Santo, Vitória, ES, Brazil; Rey Juan Carlos University: Universidad Rey Juan Carlos, SPAIN

## Abstract

The objective of this article was to analyze the factors associated with complex multimorbidity (CMM) among hemodialysis patients in a metropolitan region in southeastern Brazil. To this end, a cross-sectional epidemiological survey was carried out with 1,024 individuals in the year 2019. CMM data were collected through the application of a questionnaire to hemodialysis patients. The binary logistic regression model was used to estimate odds ratios (OR) and 95% confidence intervals (95%CI) between independent variables and CMM. The prevalence of CMM was 81% and the results indicated that: living in cities with a low rate of general mortality (OR = 0.395, 95%CI = 0.179–0.870), being aged between 18 and 29 (OR = 0.402, 95%CI = 0.196–0.825), having an elementary education (OR = 0.536, 95%CI = 0.290–0.966) and assessing health as good/very good (OR = 0.446, 95%CI = 0.301–0.661) are factors that reduced the chances of having CMM, whereas a longer period of hemodialysis (OR = 1.779 and 95%CI = 1.057–2.997) increased the chances of CMM. The findings show that characteristics of the social and individual context are associated with CMM in hemodialysis patients, signaling the need for public health policies that include monitoring the complex multimorbidity condition among individuals undergoing hemodialysis treatment.

## Introduction

The term “multimorbidity” emerged in Europe in the final half of the twentieth century [1] and, according to the World Health Organization (WHO) [2], refers to the simultaneous occurrence of two or more chronic conditions/diseases in the same individual. Interest in the study of multimorbidity has grown, especially as a result of data that show the increase both in life expectancy and in the prevalence and incidence of chronic noncommunicable diseases (NCDs) among populations [3].

Although the definition of multimorbidity proposed by the WHO is the one most widely adopted [4], there is still no consensus on this concept [5], including regarding a classification standard [6]. However, the point of convergence among authors who study the subject is the association of multimorbidity with a higher mortality rate, polypharmacy and increased use of health services, which represents an even greater challenge for health systems, especially public ones [7].

Despite the negative impacts on health, studies on multimorbidity are recent and classification methods are not very standardized, which limits the comparison of results [6]. Another limiting factor is the use of this concept in a population that is already potentially multimorbid, such as individuals in hemodialysis treatment, since they routinely present associated chronic comorbidities [8].

Although studies on multimorbidity in individuals undergoing hemodialysis are scarce, understanding this condition in this group is useful, especially in providing data to guide the therapeutic approach, allocation of resources and monitoring of individuals within the health network [9, 10].

Thus, an alternative for studying of this condition in individuals with more than two chronic conditions would be the evaluating of the occurrence of complex multimorbidity—defined by Harrison et al. [11] as the existence of three or more chronic conditions that affect three or more different organic domains (body systems)—which assesses the impairment of body systems, rather than assessing of multimorbidity simply by counting diseases, and is applicable to those individuals undergoing hemodialysis treatment.

Thus, based on the above, this study presents an innovative proposal in the analysis of complex multimorbidity in individuals undergoing hemodialysis treatment in a metropolitan region in southeastern Brazil.

## Materials and methods

This is a cross-sectional study that considered the total number of users who underwent hemodialysis in the Região Metropolitana da Grande Vitória, Espírito Santo, Brazil (RMGV-ES), from February to September 2019. The RMGV-ES aggregates the largest number of individuals undergoing hemodialysis procedures in the state of Espírito Santo.

The inclusion criteria were being 18 years of age or older, undergoing hemodialysis treatment (weekly frequency of three sessions or more lasting over two hours per session) in the RMGV-ES, walking independently without the need for assistance and having a confirmed diagnosis on the chronic kidney disease chart by the International Classification of Diseases, version 10 (ICD-10): N18 (chronic renal failure), N180 (end-stage renal disease), N188 (other chronic renal failure), N189 (unspecified chronic renal failure) and N19 (unspecified renal failure).

Chronic kidney disease was chosen as the initial criterion for analysis of other morbidities in all patients in hemodialysis treatment.

The exclusion criteria were: being hospitalized, having impaired speech and/or hearing, having physical difficulties that prevented data collection and having been transferred to receive hemodialysis in clinics located outside the RMGV-ES.

Out of the total number of individuals in the target population (1,351), 304 were excluded due to the established criteria and thus 1,047 individuals were eligible for the research. Of these, 23 (2.2%) refused to participate in the survey. Thus, the final population for the study comprised 1,024 individuals. It should be emphasized that, as this was an epidemiological census study involving the entire population undergoing hemodialysis during the study period, no sample size calculation was performed.

The data (S1 File) were collected during hemodialysis sessions via the application of a semi-structured questionnaire by interviewers trained for this purpose. The data collection instrument was prepared with validated questionnaires.

Complex multimorbidity (CMM) was characterized by the occurrence of three or more chronic conditions/diseases that affect three or more domains or different body systems [11], excluding chronic kidney disease, since having this disease was an inclusion criterion in the study.

To identify the domains/organic systems related to each condition/disease, the ICD-11 (WHO) was used with the following classification: endocrine, nutritional or metabolic diseases (dyslipidemia, diabetes, thyroid disorders); circulatory system (cardiac arrhythmia, arterial hypertension, heart attack, stroke); mental, behavioral or neurodevelopmental disorders (depression, Alzheimer’s disease); neoplasms (cancer); genitourinary system (kidney diseases—which are classified in this group, but were not included, as this was a criterion for inclusion in the study—and infertility); digestive system (liver cirrhosis); nervous system (Parkinson’s disease); pulmonary system (asthma, bronchitis, pulmonary emphysema); musculoskeletal system or connective tissue (repetitive strain injuries, work-related musculoskeletal disorders, osteoarthritis, herniated disc).

Data on the occurrence of chronic conditions/diseases were collected by the question “Has a doctor or other health professional ever told you that you had any of these diseases?”. The nontransmissible diseases/conditions investigated in this research were: arrhythmia, infarction, stroke, diabetes mellitus, disc herniation, arthrosis, Parkinson’s disease, Alzheimer’s disease, liver cirrhosis, infertility, cancer, thyroid disorders, asthma, bronchitis, pulmonary emphysema, arterial hypertension, dyslipidemia and depression. In the few cases where it was not possible to obtain information about nontransmissible diseases through self-reporting (e.g., patient did not know the answer), the medical records were consulted. In this case, the collection of patient data from medical records was carried out on the same day that the patient responded to the questionnaire.

**Fig 1:**
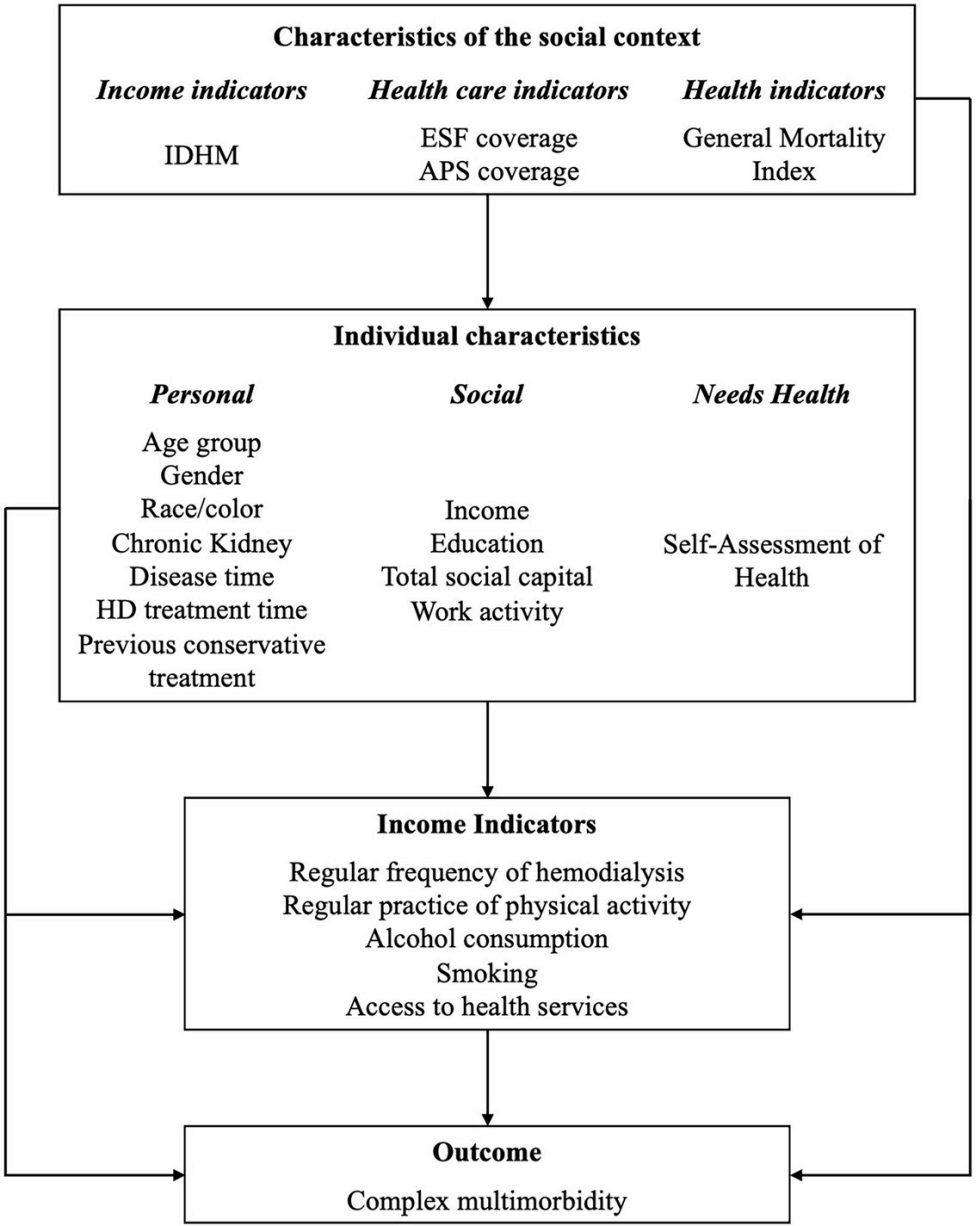
Analysis model. IDHM, Municipal Human Development Index; ESF, Family Health Strategy coverage (the gateway to public health policy in Brazil); APS, Primary Health Care (Public health policy in Brazil); HD, Hemodialysis.

The analysis model is presented in Fig 1, with the independent variables organized in blocks. The social context variables corresponded to the indicators of the patients’ municipalities of residence. The data were obtained from the Brazilian Institute of Geography and Statistics (IBGE) [12, 13] and were defined as: Municipal Human Development Index (IDHM), Coverage of the Family Health Strategy (ESF), Primary Health Care Coverage (APS) and General Mortality Index. The Municipal Human Development Index (IDHM) is an index adapted to the reality of Brazilian municipalities that summarizes social indicators referring to three dimensions related to human development: longevity, income and education. The IDHM was classificated in “very high” (above 0.800); “high” (between 0.700 and 0.800); “medium” (between 0.550 and 0.700) and “low” (below 0.550). It is noteworthy that in the RMGV, there are no municipalities classified with low IDHM. The variable “Coverage of the Family Health Strategy” (ESF) corresponds to the percentage of the municipality’s population covered by the Family Health Strategy (ESF). It was classified in (“high” defined as surpassing 75% of the population, “medium” defined as ranging between 50% and 75% of the population, and low defined as below 50% of the population). The Family Health Strategy (ESF) is a Brazilian public policy that aims to increase the effectiveness of public health services for the population. It is based on a Primary Care model of care, organized through the work of multidisciplinary teams in an assigned territory, which develops health actions based on knowledge of the local reality and the needs of its population. The variable”Primary Health Care Coverage” (APS) corresponds to the estimated population coverage of Primary Care teams in each municipality and was also categorized into “high” defined as surpassing 75% of the population, “medium” defined as ranging between 50% and 75% of the population, and “low” defined as below 50% of the population. The variable “General Mortality Index” corresponded to the Mortality Index of the municipality where the hemodialysis patient lived. The data were obtained from the Information Technology Department of the Unified Health System and classified as “> 10/1,000 inhabitants”, “5.1–10/1,000 inhabitants” and “≤ 5/1,000 inhabitants” [14].

The group of individual characteristics included personal, social and health needs variables. As personal variables, we considered: age group, gender, self-reported race/color, time on hemodialysis, previous conservative treatment (based on medication and dietary modifications, such as protein restriction), and duration of chronic kidney disease. As social variables, it was considered: income, education (primary education refers to less than 8 years of schooling, elementary education encompasses 8 to 11 years of schooling, and secondary and tertiary education encompasses 11 or more years of schooling), work activity, total social capital (these were classified according to Reisen et al. [15] into “high”, “moderate” and “low social capital”, based on the indicators proposed by Grootaert (2001) [16]. The self-assessment variable of health status was categorized into “good,” “very good”, “poor” and “very poor” and corresponded to the variable related to health needs.

In the group of variables related to “income indicators”, were include the variables: regular frequency of hemodialysis, regular practice of physical activity and variables related to health habits (practice of physical activity, alcohol consumption and smoking history, based on the CAGE questionnaire, and access to health services, classified as high, moderate and low, according to Oliveira Soares et al. [17].

To evaluate the reproducibility of the data collection instrument, a pilot test was carried out between October and December 2018 with 57 individuals undergoing hemodialysis treatment in a municipality outside the metropolitan region studied. The sample for the pilot test comprised all individuals undergoing dialysis treatment at a hemodialysis clinic located outside the geographic region of the study. These individuals were not considered for the purposes of the article’s analyses. The pilot test was divided into two parts—test and retest—with a difference of 15 days between them and with the questionnaire being applied to the same patients in both. The test and retest analyses were performed using the Kappa coefficient and McNemar’s test. WinPepi software (PEPI-for-Windows version 11.65, Hebrew University, Jerusalem, Israel) was used, and the confidence interval adopted was 95% with p < 0.05. The results of the adjusted Kappa were above 0.6, showing a high level of agreement. In the McNemar test, values of p > 0.05 were obtained, demonstrating high agreement and low disagreement, and indicating its good reproducibility.

The analysis of the research results was performed using the IBM SPSS® Statistics for Windows software, version 22.0 (Armonk, NY: IBM Corp), based on bivariate association statistics between the outcome, complex multimorbidity, and the independent variables through the Chi-square test and/or Fisher’s exact test. Model quality was assessed using the Hosmer–Lemeshow test. Missing data were disregarded from the analyzes due to low data loss. When such a situation occurred, the difference in the number of individuals in their respective variable in the tables was indicated.

Binary logistic regression analysis was performed to estimate the associations of the independent variables with the outcome—complex multimorbidity (characterized by the presence of three or more nontransmissible diseases, except for chronic kidney disease, which was the key inclusion criterion in the study), affecting three or more different organic systems. A regression model was developed using variables that exhibited a statistical significance level of α < 0.05 in the bivariate analysis results. The introduction of variables into the regression model was conducted hierarchically, in blocks. Given the hierarchical nature of the analysis, the final model accounted for the adjustment of all variables integrated into the model, specifically: Block 1 comprised income indicators; Block 2 included income indicators and individual characteristics; and Block 3 encompassed income indicators, individual characteristics, and aspects of the social context. The categories that increased the risk of developing complex multimorbidity were considered as the reference for logistic regression analysis. The stepwise variable selection method with the likelihood ratio test was used, adopting the model with the highest fit according to the Hosmer–Lemeshow test (p > 0.05, closer to 1), multicollinearity (tolerance > 0.1 and variance inflation factor < 10), minimum sample size for the number of model variables (> 20 individuals per variables in the model and > 5 cases in each category of variables) and absence of outliers (absence of standardized residuals > + 3 standard deviations, up to 1% of standardized residuals between + 2.5 and 3 standard deviations and up to 5% of standardized residuals between 2.0 and 2.5 standard deviations and/or Cook’s distance < 1 and DFBeta < 1). In the final model, only the variables that presented a significance level of α < 0.05 were maintained. It should be noted that only hemodialysis patients with responses in all variables were included in this analysis.

The research was approved by the Research Ethics Committee of the Health Sciences Center of the Federal University of Espírito Santo, under opinion number 4.023.221 and CAAE number 68528817.4.0000.5060. All hemodialysis units formally authorized the research through a letter of consent, as did all the research participants, who only started participating after reading and signing the written Informed Consent Form.

## Results

The prevalence of complex multimorbidity among patients undergoing hemodialysis treatment was 81.1% (915 individuals). The average age of those surveyed was 54.7+0.59 years old, with the predominant age group being 30 and 59 years old (n = 528, 51.6%). Most individuals (57.9%) lived in municipalities with a high IDHM, while only 388 (37.9%) lived in cities with a mortality rate of up to five deaths per 1,000 inhabitants. Most individuals were male (n = 581, 56.7%), self-declared black or brown (n = 737, 72%), with high APS coverage (n = 477, 46.6%) and a low coverage of ESF (n = 47, 46.2%).

With regard to chronic kidney disease, 525 (51.3%) had the disease for less than five years and 679 individuals (66.6%) had not undergone previous conservative treatment. As regards to hemodialysis, 368 individuals (35.9%) had been undergoing the procedure for less than three years and most individuals self-assessed their health status as good/very good (n = 633, 61.8%) (Table 1).

**Table 1:** Descriptive analysis of the variables of exposure to complex multimorbidity of hemodialysis patients, Brazil, 2019.

Variables	N	%	95%CI
**Municipal Human Development Index** (N = 1,024)			
Medium (0.550 to 0.700)	53	5.2	0.60–0.90
High (0.700 to 0.800)	593	57.9	0.74–0.82
Very high (> 0.800)	378	36.9	0.81–0.90
**Family Health Strategy Coverage** (N = 1,024)			
High (> 75% of the population)	273	26.7	0.71–0.83
Medium (50% to 75% of the population)	278	27.1	0.79–0.90
Low (< 50% of the population)	473	46.2	0.75–0.84
**Primary Health Care Coverage** (N = 1,024)			
High (> 75% of the population)	477	46.6	0.78–0.85
Medium (50% to 75% of the population)	329	32.1	0.72–0.83
Low (< 50% of the population)	218	21.3	0.73–0.86
**General Mortality Index** (N = 1,024)			
Up to 5/1,000 inhabitants	388	37.9	0.72–0.82
5.1–10/1,000 inhabitants	438	42.8	0.75–0.84
Above 10/1,000 inhabitants	198	19.3	0.83–0.95
**Age groups** (N = 1,024)			
18–29 years old	59	5,8	0,34–0,69
30–59 years old	528	51,6	0,77–0,85
60 years old and more	437	42,7	0,78–0,87
**Gender (N = 1,024)**			
Female	443	43.3	0.76–0.85
Male	581	56.7	0.75–0.83
**Race/Color (N = 1,011)**			
White	274	27.1	0.77–0.88
Black/brown	737	72.9	0.76–0.83
**Duration of Chronic Kidney Disease (N = 1,019)**			
< 5 years	525	51.5	0.75–0.83
≥ 5 years	494	48.5	0.77–0.85
**Time of Hemodialysis Treatment (N = 967)**			
0–2 years	368	38.1	0.78–0.87
3–5 years	252	26.1	0.76–0.85
6–10 years	198	20.5	0.72–0.86
Above 10 years	149	15.4	0.70–0.86
**Previous Conservative Treatment (N = 1,019)**			
No	678	66.6	0.75–0.83
Yes	341	33.4	0.78–0.87
**Monthly Income (N = 988)**			
≤ 2 minimum wages	555	56.2	0.76–0.84
> 2 minimum wages	433	43.8	0.76–0.85
**Education (N = 1,013)**			
Primary education	523	51.6	0.76–0.85
Elementary education	332	32.8	0.70–0.81
Secondary and tertiary education	158	15.6	0.82–0.95
**Total social capital (N = 988)**			
Low	273	27.6	0.78–0.89
Moderate	465	47.1	0.77–0.85
High	250	25.3	0.68–0.81
**Work Activity (N = 1,009)**			
With work activity	348	34.5	0.70–0.81
Retired or on sick leave	547	54.2	0.80–0.87
Without activity labor paid	114	11.3	0.70–0.89
**Self-Assessment of Health Status (N = 1,021)**			
Good /very good	633	62	0.71–0.79
Bad / very bad	388	38	0.84–0.92
**Alcohol Consumption (N = 1,024)**			
Without habit	929	90,7	0.77–0.84
With habit	95	9,3	0.65–0.86
**Smoking (N = 1,024)**			
Never smoked	599	58,5	0.74–0.82
Former smoker	372	36.3	0.79–0.89
Smoker Current	53	5.2	0.60–0.91
**Practice of Physical Activity (N = 1,023)**			
Within the recommended	118	11.5	0.58–079
Below recommended	111	10.9	0.67–0.87
Absence of practice	794	77.6	0.79–0.85
**Level of Access to Health Services (N = 830)**			
Low	281	33.9	0.80–0.90
Moderate	340	41	0.71–0.81
High	209	25.1	0.74–0.86

In terms of the income range, 555 (56.2%) of the hemodialysis patients had a monthly income of less than or equal to two minimum wages in force (minimum wage in force at the time of the study: R$998.00, equivalent to US$189.54). Most respondents were retired or no longer undertaking work activities and were receiving social benefits (n = 547, 54.2%), while only 209 (25.2%) enjoyed the highest level of access to health services.

In regard to total social capital, just 254 (24.9%) were categorized as having high total social capital. As for behavioral habits, only 95 (9.3%) consumed alcohol regularly, 53 (5.2%) were smokers and the vast majority (n = 794, 77.6%) did not practice physical activity (Table 1).

The results of the bivariate analyses showed that the variables IDHM (p = 0.007), General Mortality Index of the municipality of residence (p = 0.003), age group (p<0.001), sex (p = 0.037), hemodialysis duration (p = 0.013), previous conservative treatment (p = 0.043), education (p = 0.048), work activity (p = 0.047), self-assessment of health status (p<0.001) and regular practice of physical activity (p = 0.047) were related to CMM (Table 2).

**Table 2:** Presence of complex multimorbidity according to social context, individual characteristics and income indicators of hemodialysis patients, Brazil,2019.

Variables	Total		Complex multimorbidity		P-value
	N	%	Not	Yes	
**Municipal Human Development Index**					**0.007**
Medium (0.550 to 0.700)	53	5.3	14	39	
High (0.700 to 0.800)	582	57.9	124	458	
Very high (> 0.800)	370	36.8	52	318	
**Family Health Strategy Coverage**					0.069
High (> 75% of the population)	267	26.6	57	210	
Medium (50% to 75% of the population)	273	27.1	39	234	
Low (< 50% of the population)	465	46.3	94	371	
**Primary Health Care Coverage**					0.292
High (> 75% of the population)	469	46,7	79	390	
Medium (50% to 75% of the population)	322	32	66	256	
Low (< 50% of the population)	214	21,3	45	169	
**General Mortality Index**					**0.003**
> 10/1,000 inhabitants	381	37.9	88	293	
5.1–10/1,000 inhabitants	428	42.6	80	348	
≤ 5/1,000 inhabitants	196	19.5	22	174	
**Age groups**					**<0.001**
18–29 years old	55	5.5	22	33	
30–59 years old	519	51.6	100	419	
60 years old and more	431	42.9	68	363	
**Gender**					**0.037**
Female	438	43.6	70	368	
Male	567	56.4	120	447	
**Total**	1,005	100	190	815	

Chi-square test. In the descriptive analysis, all individuals with information on each of the variables were included, while in the regression analysis, only those with information available for the set of variables were included.

However, after performing binary logistic regression, the following remained in the final model and reduced the chances of CMM were: residing in a municipality with a General Mortality Index of up to five deaths per group of 1,000 inhabitants (p = 0.021; OR = 0.395; 95%CI = 0.179–0.870); belonging to the age group between of 18 and 29 years old (p = 0.013; OR = 0.402; 95%CI = 0.196–0.825); having an elementary education (p = 0.038; OR = 0.536; CI95% = 0.29–0.966); and self-assessing health status as good/very good (p<0.001; OR = 0.446; CI95% = 0.301–0.66). Undergoing hemodialysis treatment for less than two years increased the chances of CMM (p = 0.03; OR = 1.779; 95%CI = 1.057–2.997) (Table 3).

**Table 3:** Logistic regression analysis between exposure variables and complex multimorbidity among hemodialysis patients, Brazil, 2019.

Variables	Block 1	Block 1 and 2	Block 1, 2 and 3
	aOR (95%CI)	aOR (95%CI)	aOR (95%CI)
**Municipal Human Development Index**			
Medium (0.550 to 0.700)	1	1	1
High (0.700 to 0.800)	0.737 (0.320–1.693)	0.941 (0.392–2.258)	0.948 (0.396–2.270)
Very high (> 0.800)	0.897 (0.532–1.512)	0.984 (0.571–1.695)	1.000 (0.580–1.725)
**General Mortality Index**			
> 10/1,000 inhabitants	1	1	1
5.1–10/1,000 inhabitants	0.551 (0.293–1.037)	**0.510 (0.265–0.982)**	**0.506 (0.263–0.975)**
≤ 5/1,000 inhabitants	**0.448 (0.210–0.955**	**0.413 (0.188–0.906)**	**0.395 (0.179–0.870)**
**Age groups**			
18–29 years old		**0.372 (0.183–0.756)**	**0.402 (0.196–0.825)**
30–59 years old		1.157 (0.788–1.699)	1.175 (0.779–1.728)
60 years old and more		1	1
**Gender**			
Female		1	1
Male		1.145 (0.782–1.678)	1.083 (0.734–1.598)
**Time of Hemodialysis Treatment**			
0–2 years		**1.796 (1.068–3.020)**	**1.779 (1.057–2.997)**
3–5 years		1.455 (0.848–2.498)	1.447 (0.842–2.486)
6–10 years		1.179 (0.679–2.048)	1.175 (0.675–2.043)
Above 10 years		1	1
**Previous Conservative Treatment**			
No			0.777 (0.527–1.146)
Yes			1
**Education**			
Secondary and tertiary education	0.584 (0.324–1.052)	0.567 (0.314–1.024)	
Elementary education	**0.550 (0.305–0.989)**	**0.536 (0.297–0.966)**	
Primary education	1	1	
**Work Activity**			
With work activity		0.680 (0.357–1.295)	0.674 (0.353–1.287)
Retired or on sick leave		1.169 (0.628–2.173)	1.157 (0.622–2.153)
Without activity labor paid		1	1
**Self-Assessment of Health Status**			
Bad / very bad		1	1
Good /very good		**0.435 (0.294–0.643)**	**0.446 (0.31–0.661)**
**Practice of Physical Activity**			
Within the recommended			0.651 (0.384–1.104)
Below recommended			0.800 (0.471–1.358)
Absence of practice			1

Binary logistic regression. Variables demonstrating a statistical significance level of α < 0.05 in the outcomes of the bivariate analyses were incorporated into the adjustment of the binary logistic regression model. The inclusion of variables into the regression model was structured in stages: Block 1 encompassed income indicators; Block 2 included both income indicators and individual characteristics; and Block 3 comprised income indicators, individual characteristics, and aspects of the social context. Only those with information available for the set of variables were included in the regression analysis.

## Discussion

This research presents an unprecedented proposal in addressing complex multimorbidity in potentially multimorbid individuals. The findings showed that the prevalence of CMM among individuals undergoing hemodialysis during the study period was 81.1%. This result can be analyzed in relation to the etiological characteristic of chronic kidney disease, as a consequence of other nontransmissible diseases, such as diabetes mellitus and systemic arterial hypertension [16, 18].

When analyzing the variables of the social context according to the proposed analysis model, it was found that the lower general mortality rates of the individuals’ city of residence reduced the chances of CMM.

These data can be analyzed from the perspective of the proposition presented by Krieger [19] when associating the social context of individuals’ lives with the health conditions presented by them. This author proposes a cause-and-effect relationship between social context and health conditions, reinforcing the need to promote public health actions that reduce inequalities [20, 21], since the general mortality rate indirectly reflects the health conditions in which individuals reside. Although there are studies, like this one, that indicate that better social conditions are associated with a lower prevalence of multimorbidity [22], it is necessary to recognize that the present work is pioneering in the investigation of the influence of social context variables on the development of complex multimorbidity in hemodialysis patients.

With regard to individual characteristics, it appears that belonging to the younger age group and having received more than eight years of schooling reduce the chances of CMM by 60% and 47%, respectively. These findings reinforce data that indicate a greater risk of developing nontransmissible diseases among older individuals with lower levels of education, although the results of this study regarding the level of education did not show a significant difference between elementary and secondary and tertiary education levels [23–27].

The Technical Series on Safer Primary Care from the World Health Organization [28] highlights the need for monitoring health among older individuals, since the prevalence of multimorbidity in this group is higher than that among younger people. In this sense, the results of this study, unprecedented in the approach to complex multimorbidity in hemodialysis patients, can subsidize the actions of programs and public health policies that aimed at managing the organization of services in the face of the challenge of the growing prevalence of multimorbidity.

In Brazil, the development of health policies focusing on primary health care has made it possible to control diseases such as high blood pressure and diabetes mellitus, which are risk factors for chronic kidney disease [29, 30]. Another aspect to be highlighted is the regionalization of health, which allows a contextualized approach to health education actions developed within the scope of primary health care, in accordance with identified local priorities. This organizational model allows for greater approximation among hemodialysis patients and can be consolidated as an effective strategy for promoting education and health, although the qualification of care for individuals undergoing hemodialysis treatment in primary health care is still being implemented [31–33].

The data from this article indicate that positive self-perception of health status is associated with better effective health conditions. The self-assessment of health status has been configured as a important marker of functional disability [34], widely used in epidemiological surveys due to its predictive power on mortality, morbidity and the use of health services. Due to its reliability and validity being equivalent to other more complex assessments of health conditions, self-assessment of health status should be considered an important complement to objective measures [35–37]. The results referring to the duration of hemodialysis treatment indicate that individuals undergoing treatments initiated less than two years prior to data collectionare were 77% more likely to experience complex multimorbidity than those have been receiving hemodialysis above ten years. This finding may reflect adaptation to the characteristics of weekly and continuous treatment, and be analyzed from the perspective of the impact that the diagnosis of chronic kidney disease in the end stage has on the individual. Not infrequently, the first reactions to the need for routine adjustment are nonacceptance of the disease and consequent treatment, which may lead to clinical complications associated with CMM [17].

Another aspect to be considered regarding these data (association of shorter hemodialysis treatment time with a greater chance of occurrence of CMM) is the time of diagnosis of chronic kidney disease, since most of these individuals were diagnosed when the disease was already in the terminal phase, and therefore several of their organic systems were already impaired [35].

Although the variable “practice of physical activity” was not maintained in the final logistic regression model, it was associated with the occurrence of CMM in the association analyses and cannot be disregarded as an instrument for health promotion and disease prevention [36–40]. Fukushima, Costa and Orlandi [41], in studying the physical activity level of chronic kidney disease patients undergoing hemodialysis, found that most individuals were insufficiently active, which may reflect the treatment routine and concern over fistula maintenance [41–44]. However, Da Cunha et al. [45], in researching a similar population, found no association between leisure practices and clinical and treatment characteristics, indicating the need to strengthen public policy actions that allow greater access to physical activity by this population. Although there are no data to support the association between inadequate practice of physical activity and complex multimorbidity, Vancampfort et al. [46], in studying a population of low-income countries, found an association between multimorbidity and low practice of physical activity.

At this point, it is pertinent to highlight the novelty of this study’s proposal, when evaluating CMM in patients with CKD undergoing hemodialysis treatment. The results found indicating a high prevalence of CMM, signal practical implications in the health care of these patients. The clinical management of patients with CKD on hemodialysis with CMM becomes more challenging, especially in coordinating care, choosing appropriate therapies and managing drug interactions. This requires a multidisciplinary, integrated approach to care and an in-depth understanding of each patient’s individual needs [2, 3, 12, 21, 27].

Another aspect to be considered is the increased costs for treating CMM in individuals on hemodialysis. In this sense, the greater financial pressure on health systems as a result of the care demanded, implies the need for a budget planning system focused on the efficiency of resource allocation and management [24]. The management of CMM in patients with CKD undergoing hemodialysis also requires a patient-centered approach and the practice of effective health communication to optimize clinical results, which highlights the need to adopt clinical protocols adapted to individual needs [12, 25, 26].

Additional practical implication that must be highlighted from the results found is related to the fact that to mitigate the high prevalence of complex multimorbidity in hemodialysis patients, it is essential to strengthen public policies with a renewed focus on primary and secondary health prevention, highlighting effective education actions in health [31–33].

The high prevalence of complex multimorbidity in patients undergoing hemodialysis identified in this study reveals a scenario that is still little known. The identification of the association of factors such as age group, duration of hemodialysis, self-assessment of health status and general mortality rate with MMC among patients undergoing hemodialysis treatment, enables the adoption of targeted clinical therapeutic approaches in the management of these patients, adapting the treatment protocols and promoting specific preventive interventions. Furthermore, early identification of patients at higher risk can facilitate the implementation of coordinated and multidisciplinary care strategies, aiming to reduce morbidity and mortality in this vulnerable population, thus improving the effectiveness and efficiency of public health services aimed at hemodialysis patients [12, 24, 26, 31].

Finally, the urgent need to identifying CMM data for organizing of the health system is highlighted, since this condition will lead, as a possible consequence, to an increase in the demand for highly complex services. Therefore, propositions about strengthening primary health care [25, 26] seem to be a viable way to reduce the distance between patients with chronic kidney disease and health services, in addition to reducing the costs required to treat the disease and increasing the possibility of investments in health promotion actions.

Regarding the limitations of this study, it is necessary to recognize that data collection on diseases was carried out based on participants’ self-report, which, although it is a valid methodology for the study of CMM, can lead to underreporting due to insufficient knowledge or even to information bias among the individuals involved. It is worth mentioning that, to improve the internal validity of the study, information from medical records was also consulted to evaluate existing diseases, with consequent mitigation of this bias.

Another aspect to be highlighted is the cross-sectional nature of the study, in which the dialogical relationship between the exposure and outcome variables needs to be considered, such as the possibility of reverse causality, especially between the variables general mortality rate of the municipality of residence and self-assessment of health status with MMC. However, to mitigate this effect and control possible confounding factors, control variables on contextual characteristics and individual characteristics were included in the analyses, in addition to the use of robust statistical approaches. The appropriate statistical treatment of these variables allowed a more precise and reliable analysis of the factors associated with MMC in patients undergoing hemodialysis treatment, as described in the Materials and Methods section.

Another limitation that deserves to be highlighted is the fact that in this study it was not possible to identify the cause of nephropathy. Therefore, patients who reported diabetes and hypertension may have presented these conditions as a cause of nephropathy and not as a consequence of kidney disease, as in the theoretical model proposed by this study.

However, the absence of a single standard for the scientific assessment of multimorbidity, combined with the lack of research on the topic involving patients with chronic kidney disease undergoing hemodialysis, limited the comparison of results. We conclude that the prevalence of complex multimorbidity is high in patients with chronic kidney disease undergoing hemodialysis treatment. This indicates that, in addition to hemodialysis treatment, these individuals demand other health-care services of medium and high complexity with assistance from multidisciplinary teams.

The factors that reduced the chances of the occurrence of CMM among chronic kidney disease patients on hemodialysis were: a low mortality rate in the municipality of residence; aged group between 18 and 29 years; positive self-rated health; and eight years or more of schooling, thus reinforcing the role of contextual and individual characteristics.

On the other hand, treatment for less than two years was a factor that increased the chances of CMM, which indicates the need for monitoring health, especially for individuals who have a clinical condition at risk of chronic kidney disease. Thus, public health policies that promote effective health education actions, follow-up of individuals under continuous treatment, such as hemodialysis, within the health-care network, and monitoring of multimorbid individuals are presented as viable public policy actions in the approach to complex multimorbidity in hemodialysis patients.

## Supporting information

S1 File Minimal data set. (XLSX)
